# Epidemic History and Evolutionary Dynamics of Hepatitis B Virus Infection in Two Remote Communities in Rural Nigeria

**DOI:** 10.1371/journal.pone.0011615

**Published:** 2010-07-19

**Authors:** Joseph C. Forbi, Gilberto Vaughan, Michael A. Purdy, David S. Campo, Guo-liang Xia, Lilia M. Ganova-Raeva, Sumathi Ramachandran, Hong Thai, Yury E. Khudyakov

**Affiliations:** Division of Viral Hepatitis, Centers for Disease Control and Prevention, Atlanta, Georgia, United States of America; Singapore Institute for Clinical Sciences, Singapore

## Abstract

**Background:**

In Nigeria, hepatitis B virus (HBV) infection has reached hyperendemic levels and its nature and origin have been described as a puzzle. In this study, we investigated the molecular epidemiology and epidemic history of HBV infection in two semi-isolated rural communities in North/Central Nigeria. It was expected that only a few, if any, HBV strains could have been introduced and effectively transmitted among these residents, reflecting limited contacts of these communities with the general population in the country.

**Methods and Findings:**

Despite remoteness and isolation, ∼11% of the entire population in these communities was HBV-DNA seropositive. Analyses of the *S*-gene sequences obtained from 55 HBV-seropositive individuals showed the circulation of 37 distinct HBV variants. These HBV isolates belong predominantly to genotype E (HBV/E) (n = 53, 96.4%), with only 2 classified as sub-genotype A3 (HBV/A3). Phylogenetic analysis showed extensive intermixing between HBV/E variants identified in these communities and different countries in Africa. Quasispecies analysis of 22 HBV/E strains using end-point limiting-dilution real-time PCR, sequencing and median joining networks showed extensive intra-host heterogeneity and inter-host variant sharing. To investigate events that resulted in such remarkable HBV/E diversity, HBV full-size genome sequences were obtained from 47 HBV/E infected persons and *P* gene was subjected to Bayesian coalescent analysis. The time to the most recent common ancestor (tMRCA) for these HBV/E variants was estimated to be year 1952 (95% highest posterior density (95% HPD): 1927–1970). Using additional HBV/E sequences from other African countries, the tMRCA was estimated to be year 1948 (95% HPD: 1924–1966), indicating that HBV/E in these remote communities has a similar time of origin with multiple HBV/E variants broadly circulating in West/Central Africa. Phylogenetic analysis and statistical neutrality tests suggested rapid HBV/E population expansion. Additionally, skyline plot analysis showed an increase in the size of the HBV/E-infected population over the last ∼30–40 years.

**Conclusions:**

Our data suggest a massive introduction and relatively recent HBV/E expansion in the human population in Africa. Collectively, these data show a significant shift in the HBV/E epidemic dynamics in Africa over the last century.

## Introduction 

Hepatitis B virus (HBV) infection presents a global health problem. Worldwide, at least 2 billion people or one third of the world's population have been infected with HBV. Approximately 378 million people are chronic carriers and ∼620,000 people die each year from acute and chronic sequelae of HBV infection [1], [2]. Furthermore, 4.5 million new HBV infections occur worldwide each year, of which a quarter progress to liver disease [3].

The HBV genome is a partially double-stranded circular DNA molecule of ∼3.2kb in length. It contains four partly overlapping open reading frames (ORFs): the preS1/preS2/S ORF encoding the hepatitis B surface antigen (HBsAg); the precore/core ORF encoding the hepatitis B e antigen and core protein; the P ORF encoding the polymerase protein; and the X ORF encoding the X protein [4]. The HBV genome is heterogeneous and can be classified into 8 genotypes: A, B, C, D, E, F, G and H. Currently, HBV genotypes are defined based on at least 8% divergence across the complete genome sequence [5] and less than 4% intra-genotypic divergence [6].

HBV genotypes are known to be geographically segregated [7]. Genotype E (HBV/E) is predominant in the West/Central African crescent spanning from Senegal to Angola [8], [9], [10]. Despite the extensive spread of HBV/E in this crescent, the virus is marked by a peculiarly low overall sequence diversity of 1.75% over the whole genome [8]. It has been suggested that it would take 200 years to generate the current extent of HBV/E diversity [11]. Rare findings of HBV/E in the Americas suggest a relatively recent HBV/E expansion in the human population in Africa, because the high HBV/E prevalence in Africa coupled with the high volume of the Afro-American slave trade that took place between the 17^th^ and the 19^th^ century [8], [11], [12], [13] would have resulted in frequent detection of HBV/E infection in the Americas. How HBV/E may have spread within and throughout the HBV/E crescent remains a puzzle.

In Nigeria, HBV infection has reached hyperendemic levels with the seroprevalence of HBsAg estimated to range from 10–40% [13], [14], [15], [16]. Despite the availability of a safe and effective vaccine since 1982 [17], [18] and its inclusion in Nigeria's national immunization program in 1995, the vaccine became available to the country only in 2004 [19], [20]. Owing to this late entry and the absence of a national HBV surveillance program, the burden of hepatitis B remains substantial.

The present study was conducted to obtain insight into the molecular epidemiology of HBV/E in two remote rural communities in Nigeria, West Africa. These communities have limited contact with the general population in the country. It was expected that they were shielded from the major epidemiological processes that introduced HBV infection in Nigeria. Sub-genomic and whole-genome-based molecular epidemiological investigation identified multifarious HBV/E strains in these communities. Evolutionary analyses indicated the recent origin of the modern HBV/E variants and suggested a massive introduction of HBV to the communities at the same time it was introduced to the entire West/Central Africa sub-region.

## Materials and Methods

### Serum samples and HBsAg serology

A total of 500 serum samples were collected from asymptomatic volunteers residing in two neighboring remote rural communities in Nasarawa State, Nigeria, West Africa in 2007. These two remote communities are ∼2 miles from each other with a total population of ∼2000 inhabitants. The traditional heads of the communities served as the society entry points. Written informed consent was obtained from all participants involved in this study. The use of specimens in this study was approved by the Institutional Review Board of the Centers for Disease Control and Prevention, Atlanta, GA and the Nigerian Federal ministry of Health. The inhabitants of these villages are of low socio-economic status lacking basic medical facilities. The commercially available Smart Check immunoassay (Globalemed, Cape Town, South Africa) for the qualitative detection of HBsAg by rapid chromatography was used for screening HBV infection in all the samples and was conducted according to the manufacturer's instructions.

### Nucleic acid extraction

Total nucleic acid was isolated from all serum samples (n = 500) using the robotic Roche MagNA Pure LC system (software version 3.0.11) and the MagNA Pure LC Total Nucleic acid isolation kit (Roche, Applied Sciences, Indianapolis, IN) according to manufacturer's instructions.

### PCR amplification of the *S* gene

Amplification of the partial *S*-gene was performed in a nested format to yield a product of approximately 400 bp using: for first-round PCR primer pairs, HBV_S1F (position 179), 5′-CGA TTT AGG TGA CAC TAT AGA AGA GAG GCT CTA GGA CCC CTG CTC GTG TT, and HBV_S1R (position 704), 5′-CAG TAA TAC GAC TCA CTA TAG GGA GAA GGC TCG AAC CAC TGA ACA AAT GGC ACT; and for second-round PCR primer pairs, HBV_SNF (position 217), 5′-CGA TTT AGG TGA CAC TAT AGA AGA GAG GCT GTT GAC AAG AAT CCT CAC AAT ACC, and HBV_SNR (position 658), 5′-CAG TAA TAC GAC TCA CTA TAG GGA GAA GGC GGC TGA GGC CCA CTC CCA TA. After a denaturation step at 95°C for 10 min, PCR reactions were performed in 30 cycles (95°C for 30 sec, 55°C for 1 min and 72°C for 32 sec) followed by melting curve analysis (95°C for 1 min, 80°C for 30 sec and 95°C for 30 sec) performed in one cycle using an Mx3005P SYBR Green Real-Time PCR System (Stratagene, La Jolla, CA).

### HBV whole-genome amplification

Full-length HBV genome was amplified by two rounds of PCR. The first round of PCR was conducted using the primer combination of HBV1798FLong and HBV1801RLong (Table 1) as follows: initial denaturation (94°C for 3 min), followed by 10 cycles with each cycle consisting of denaturation at 94°C for 20 sec, annealing at 55°C −45°C for 30 sec, and extension at 68°C for 4 min. Annealing temperature was reduced at the rate of 1°C per cycle from 55°C to 45°C. Thereafter followed 35 cycles, with each cycle consisting of denaturation at 94°C for 20 sec, annealing at 45°C for 30 sec and extension at 68°C for 4 min. The extension time was increased by 10 sec/cycle up to 7.2 min. The final extension was at 68°C for 10 min followed by cooling at 4°C. The first-round PCR was performed on the GeneAmp® PCR system 9700 (Applied Biosystem) using the Expand High-Fidelity PCR test kit. The nested PCR was done with set of six overlapping fragments, using the following primer combinations (Table 1): (HBV1847FS with HBV2394RS, HBV2298FS with HBV2933RS, HBV2821FS with HBV0272RS, HBV0179FS with HBV0704RS, HBV0599FS with HBV1286RS and HBV1175FS with HBV1788RS) under the following cycling conditions: pre-incubation at 95°C for 5 min, after which amplification for 25 cycles, each cycle consisting of denaturation at 95°C for 15 sec, annealing at 55°C for 20 sec and extension at 72°C for 1 min. After amplification, melting curve analysis was performed by raising the temperature to 95°C for 0.01 sec in one cycle using the Mx3005P SYBR Green Real-Time PCR System (Stratagene, La Jolla, CA). The derivative melting curves were obtained with the instrument data analysis software.

**Table 1:** PCR primers.

Primer name	Primer sequence
HBV1798FLong	CTGCGCACCAGCACCATGCAACTTTTTC
HBV1801RLong	CAGACCAATTTATGCCTACAGCCTCCTA
HBV1847FS	CGATTTAGGTGACACTATAGAAGAGAGGCTTGTTCATGTCCCACTGTTCAA
HBV2394RS	CAGTAATACGACTCACTATAGGGAGAAGGCTGGCGAGGGAGTTCTT
HBV2298FS	CGATTTAGGTGACACTATAGAAGAGAGGCTGACCACCAAATGCCCCTAT
HBV2933RS	CAGTAATACGACTCACTATAGGGAGAAGGCTTCGGGAAAGAATCCCAGAGGAT
HBV2821FS	CGATTTAGGTGACACTATAGAAGAGAGGCTGGTCACCATATTCTTGGGAAC
HBV0272RS	CAGTAATACGACTCACTATAGGGAGAAGGCTTGAGAGAAGTCCACCACGAGT
HBV0179FS	CGATTTAGGTGACACTATAGAAGAGAGGCTCTAGGACCCCTGCTCGTGTT
HBV0704RS	CAGTAATACGACTCACTATAGGGAGAAGGCTCGAACCACTGAACAAATGGCACT
HBV0599FS	CGATTTAGGTGACACTATAGAAGAGAGGCTGTATTCCCATCCCATCATCCTG
HBV1286RS	CAGTAATACGACTCACTATAGGGAGAAGGCTGCTAGGAGTTCCGCAGTATGG
HBV1175FS	CGATTTAGGTGACACTATAGAAGAGAGGCTGCCAAGTGTTTGCTGA
HBV1788RS	CAGTAATACGACTCACTATAGGGAGAAGGCTGCCTACAGCCTCCTA
SP6	cgatttaggtgacactatagaagagaggct
T7	cagtaatacgactcactatagggagaaggct

*Numbers within primer names represent the primer positions. An F after the primer position stands for sense primers while R stands for anti-sense primers.

**SP6 and T7 are tag sequences attached at the 5′ end of all PCR primers used in this study. SP6 and T7 primers were used to sequence PCR fragments.

### *S*-gene quasispecies analysis

Twenty-two HBV/E samples representing the major branches of the HBV/E phylogenetic tree and the two HBV/A3 samples were used for quasispecies analysis. Five specimens in this HBV/E panel were obtained from 2 groups of siblings. Additionally, when selecting samples for quasispecies analysis, preference was given to genetically close HBV/E variants identified in each major branch of the phylogenetic tree in order to assess potential transmissions. This subset was analyzed using an adaptation of our previous method, end-point limiting-dilution real-time PCR (EPLD-PCR) to HBV [21] that could detect HBV *S*-gene variants at a concentration of 0.1% of the total viral population [22]. Briefly, EPLD-PCR was performed using serially diluted DNA. The Biomek® 3000 robotic work station (Beckman Coulter, Brea, CA) was used to obtain 0.25 log dilutions of the HBV DNA. The dilution that resulted in positivity in two of four replicates was considered to be limiting (DNA target templates being assumed to be distributed in a Poisson manner so that 50% or less reactions do not carry template molecules and thus do not generate PCR products). Under such conditions, the positive reactions are most likely to have been initiated from a single template molecule. The LightCycler® 480 software (Version 1.5.0.SP3, Roche, Indianapolis, IN,) was used to perform the melting curve analysis. For each isolate, 96 EPLD amplification reactions were carried out to obtain approximately 48 clones per sample; the exact number varied depending on the viral titer. The PerfeCTa SYBR FastMix chemistry (Quanta BioSciences, Gaithersburg, MD) was used for the PCR amplification.

### DNA sequencing

Sequencing was performed using the second-round PCR products. SP6 and T7 primers (Table 1) were used for sequencing of the PCR products using BigDye version 3.1 in an automated DNA sequencer ABI 3130xl (Applied Biosystems, Foster City, CA). Sequencing PCR involved 25 cycles, each cycle consisting of 96°C for 10 sec, 50°C for 5 sec and 60°C for 4 min. Sequence electrophoregrams were initially analyzed and edited using the SeqMan and MegAlign programs of the Lasergene DNA and protein software version 7.0 (DNASTAR Inc., Madison, WI).

### Phylogenetic analysis and inference of serotypes

Nucleotide sequences were aligned using the GCG (Version 11.1.2-UNIX, Accelrys Software Inc, San Diego, CA) multiple alignment program Pileup. HBV genotypes were classified based on the *S*-gene sequence and confirmed with whole-genome sequences by comparing each sequence with published reference sequences from GenBank. Initial Neighbor-joining trees were built using the Kimura two-parameter model of nucleotide substitution [23]. Phylogenetic trees were constructed using the maximum likelihood algorithm implemented in Dnaml (PHYLIP package, v.3.6). Frequency distributions of pair-wise distances between nucleotide sequences were estimated using the evolution program in the Accelrys GCG Package (Genetic Computer Group, version 11.1-UNIX, Accelrys Inc., San Deigo, CA). SAS for Windows (Version 9.12, SAS Institute Inc., Cary, NC) was used for statistical analysis. HBV serotypes were predicted based on amino acid sequences at positions 122, 160, 127 and 134 in the *S* gene [24], [25].

### Median-Joining network (MJN)

MJN of the *S*-gene quasispecies of HBV/E and HBV/A3 were constructed using the program NETWORK 4.0 [26]. The MJN method begins computing the minimum spanning trees (a graph that connects all the sequences with the minimum necessary total length of the branches), following which all the constructed graphs are combined within a single (reticulate) network. Aiming at parsimony, the algorithm subsequently adds a few consensus sequences (called Median Vectors) of three mutually close sequences at a time. These median vectors can be biologically interpreted as possibly extant but unsampled sequences or extinct ancestral sequences. The resulting network normally harbors all optimal trees, as well as numerous suboptimal trees [26].

### Genetic structure of HBV/E

Every complete genome of HBV was obtained from the National Center for Biotechnology Information Website (NCBI) in late 2008. All sequences were aligned using ClustalW [27]. Further refinement of the alignment was made manually. Two tests for detecting population growth based on the Tajima's D [28] and Fu's F_S_ [29] statistics were performed using ARLEQUIN [30]. A p value of 0.05 or less was considered statistically significant.

### Geographic distribution

Nucleotide sequences identical to the most frequent *S*- gene sequence variant found in this study were selected from GenBank using BLAST [31]. Information on the geographic location where these sequences were identified was extracted from GenBank and published articles.

### Prediction of the Most Recent Common Ancestor (MRCA)

MRCA of the *S* gene was determined using FASTML version 2.02 [32] from the basal node on a maximum-likelihood tree of nucleotide sequences deduced from the *S*-gene quasispecies of 22 HBV/E variants, using as out-group a sequence from a patient infected by a different genotype.

### Divergence time calculations of HBV genotype E

Divergence times were calculated using 47 *P* gene sequences of HBV/E from the two Nigerian villages. A second sequence alignment was created by adding 12 sequences from GenBank with known dates of collection to the original Nigerian sequences. The accession numbers for these sequences are (AB194947), (AB194948), (AB205129), (AB205189), (AB205190), (AB205191), (AB205192), (AP007262), (AY738144), (AY738145), (AY738146) and (AY738147). Divergence times were calculated using BEAST v 1.4.8 [33]. The GTR substitution model was used with four gamma categories and invariant sites. Codons were partitioned into three with unlinked substitution model and unlinked rate heterogeneity model across the codon partitions. Each sequence alignment was analyzed with a strict or relaxed clock with an initial estimate for the rate of substitution as 2.97×10^−4^ [34]. Constant size, exponential and expansion growth priors were used. All models were run until the effective sample size for each was greater than 200. In order to compare the models, the Bayes Factor was estimated using importance sampling of the posterior probability [35]. The exponential substitution model with an expansion growth prior was chosen because it had the largest Bayes Factor; however, none of the models tested was found to be superior to any other model. Only HBV/E isolates were included in the BEAST analysis.

The effective population of the Nigerian HBV sequences was calculated using the skyline plot as implemented in BEAST. All models tested showed an expansion in the effective population of HBV in these two villages. The Bayes Factor for these models indicated that no single model was superior to any other model. The plot chosen for presentation here was the strict clock with the piecewise linear Bayesian skyline model with three population groups.

### Detection of positive selection

To determine if the HBV/E strains that are uniquely restricted to the HBV/E crescent are adapted to the populations in that region, we calculated overall and site-by-site selection pressures acting on the four ORFs in the 47-HBV/E whole genomes. We estimated the mean numbers of non-synonymous substitutions (*d*_N_) and synonymous substitutions (*d*_S_) per site (ratio d_N_/d_S_) using the Fixed Effects Likelihood (FEL) analysis implemented in the program HyPhy 0.99 beta [36], which is available in a parallel computing fashion at the Datamonkey web interface [37]. The algorithm works in three phases [38], [39]: first, the General Time Reversible nucleotide model was fitted to the data and tree using maximum likelihood to obtain branch lengths and substitution rates; second, a codon model was fitted to the data to obtain codon branch lengths for scaling *d*_N_ and d_S_ estimated subsequently from each site; and thirdly, a site-by-site likelihood-ratio test was performed to assess whether *d*_N_ is significantly different from *d*_S_.

### Nucleotide sequence accession numbers

The *S*-gene sequences of the 55 HBV isolates have been deposited in the National Center for Biotechnology Information GenBank database under the accession numbers (HM363565) to (HM363619). The 47 HBV/E and the two HBV/A3 full-genome sequences were deposited with accession numbers (HM363565) to (HM363611) and (HM363612) to (HM363613) respectively.

## Results

### HBV seroprevalence

The 500 samples collected from the 2 villages were tested for HBsAg and for HBV DNA by the *S*-gene PCR. Fifty-three samples were HBsAg-positive and 55 were HBV-DNA-positive. All HBsAg-positive specimens were found to be HBV DNA-positive. However, HBsAg was not detected in 2 HBV DNA-positive specimens. In all, 55 samples were HBV-positive giving an overall prevalence of 11%, indicating a high prevalence of HBV infection.

### Phylogenetic analysis of HBV variants

**Figure 1:**
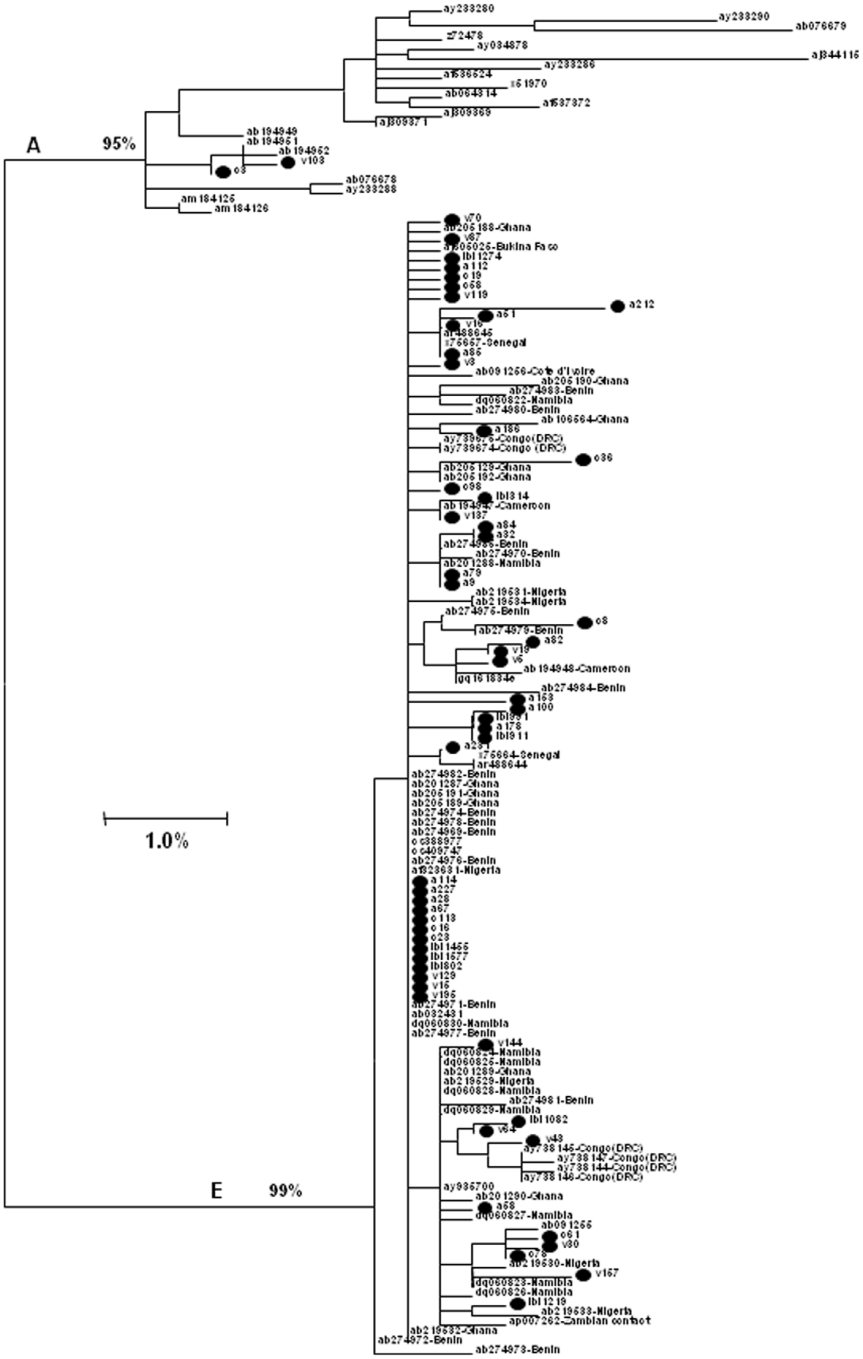
Phylogenetic tree of the partial S-gene sequences (378bp) from 55 Nigerian HBV isolates identified in this study (marked •) and sequences recovered from GenBank (not marked). Sequences retrieved from GenBank are denoted by their accession numbers and the source country of the isolates. Bootstrap values of major branches are shown.

**Figure 2:**
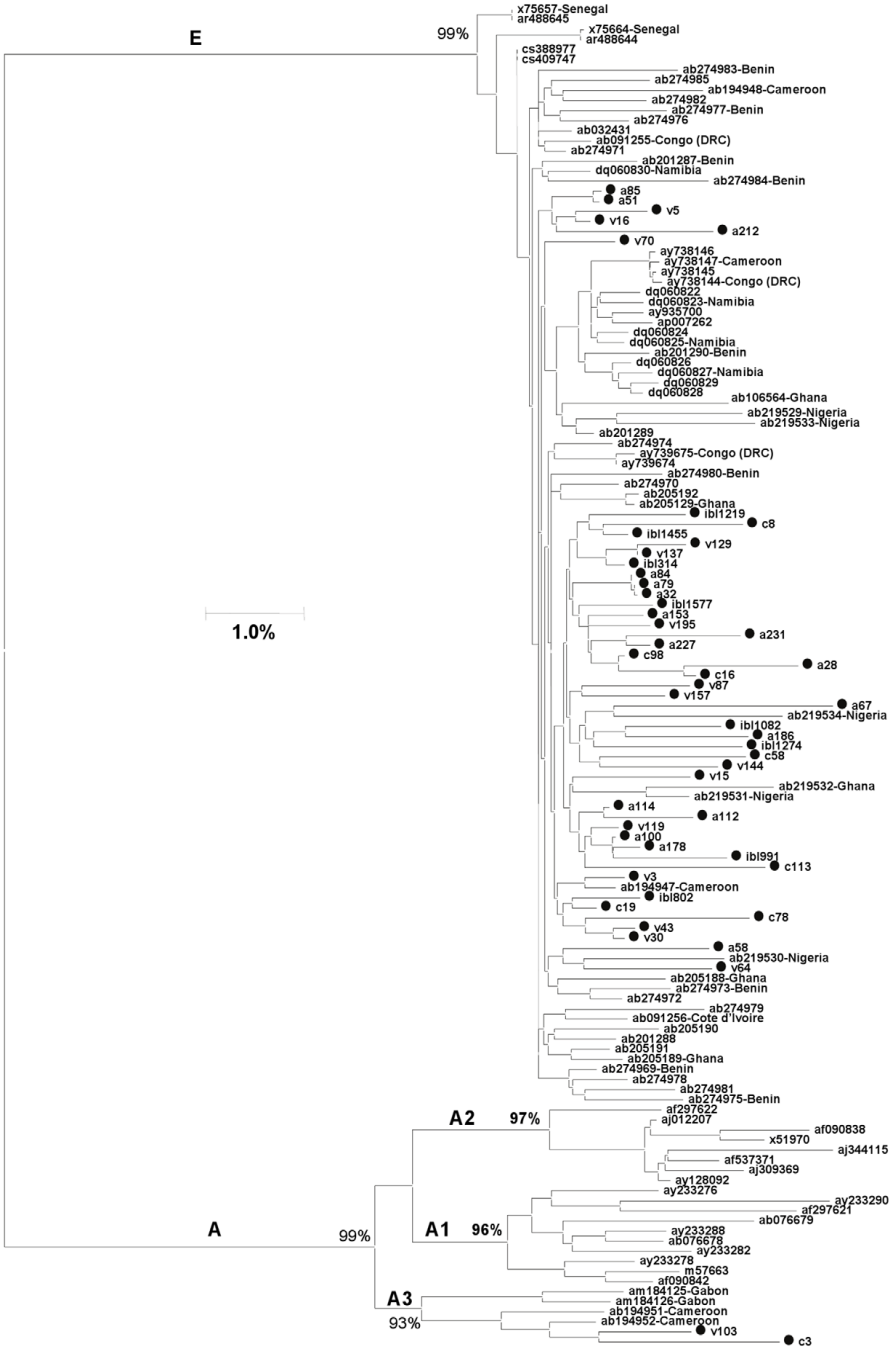
Phylogenetic tree of the 47 HBV/E and 2-HBV/A3 whole genome sequences from Nigeria (marked •) and GenBank references (not marked). Sequences retrieved from GenBank are denoted by their accession numbers and the source country of the isolates. Bootstrap values of major branches are shown.

Taking into consideration the relative isolation of these 2 communities, it can be expected that only a single HBV strain or very few strains are in circulation in their populations. However, analysis of the *S*-gene sequences identified 2 HBV genotypes, A (sub-genotype A3, n = 2) and E (n = 53), with 2 and 35 distinct sequence variants representing HBV sub-genotype A3 and genotype E, respectively (Fig. 1). To further characterize these HBV variants, the full-length HBV-genome sequences from 47 HBV/E-infected individuals and 2 HBV/A3-infected individuals were obtained. Six HBV/E isolates did not yield a complete sequence probably due to low viral titers. Phylogenetic analysis of the full genomes for 47 HBV/E and two A3 isolates confirmed the genotype classification based on the *S*-gene sequences (Fig. 2) and further substantiated that a wide diversity of HBV strains circulated in both communities. Consistent with analysis of the *S*-gene sequences, phylogenetic analysis of the whole-genome sequences (Fig. 2) did not reveal any village-specific clustering and showed extensive intermixing of these sequences with other HBV/E variants identified in different countries in Africa.

### Serotype distribution of HBV strains

Based on the presence of Arg^122^, Lys^160^ and Leu^127^/Ile^127^ [24], [25] all the A3 isolates could be classified as serotype *ayw1*. Among the 53 HBV/E isolates, two isolates could be classified as serotype *ayw2* (3.7%). All the others belonged to *ayw4* (96.3%). The *ayw2* was deduced by the presence of Pro^127^ and Phe^134^ [24], [25].

### Intra-host HBV heterogeneity

**Figure 3:**
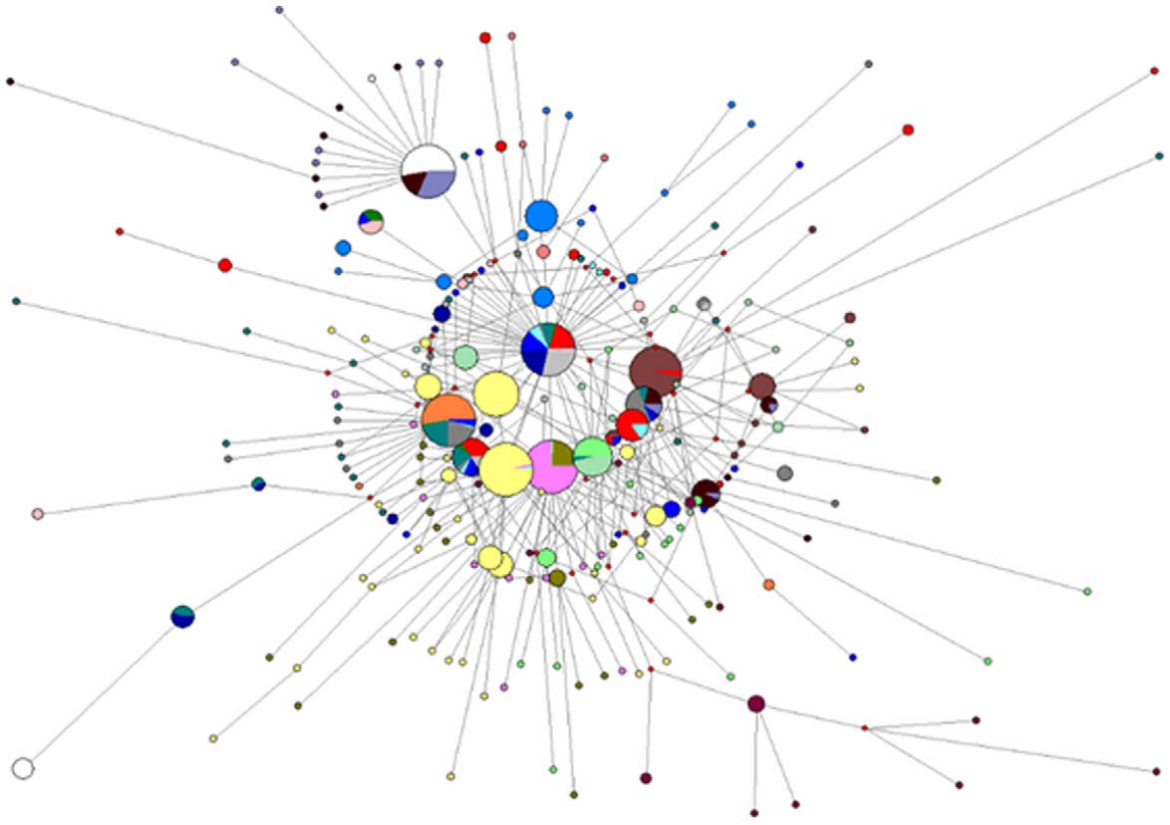
Median joining network of the intra-host S-gene sequence variants identified in 22 individuals infected with HBV/E. Each node represents a single sequence variant. Each color represents a single individual. The size of the node reflects frequency of the corresponding variant in the population.

**Figure 4:**
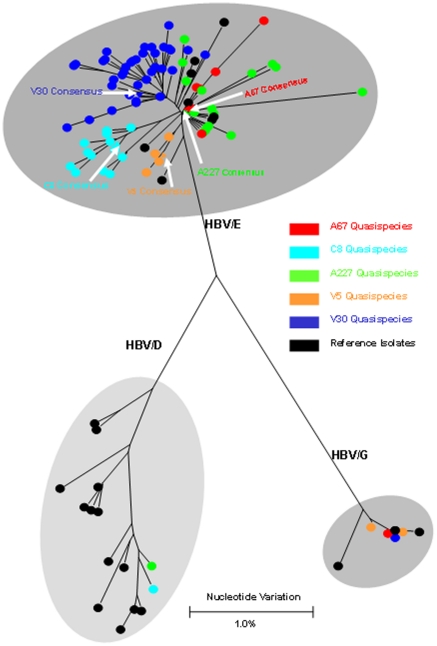
Phylogenetic tree of HBV sequence variants identified in 5 individuals with mixed genotype infections. Sequences identified in this study are colored. Reference sequences retrieved from GenBank for genotypes HBV/E, HBV/G and HBV/D are shown in black. The consensus S-gene sequences for each individual are indicated with white arrows. All sequences that obtained from a single individual are shown using same color.

The presence of 2 HBV genotypes and many HBV strains raises questions about the origin and maintenance of this remarkable HBV diversity in these two communities. To investigate the extent of variant/strain distribution and their spread within the population, quasispecies analysis was conducted using EPLD RT-PCR [21] on 2 HBV/A3 and 22 HBV/E specimens. The quasispecies analysis revealed many HBV/E variants (n = 229) circulating in these 2 communities and substantial intra-host diversity in all individuals (Fig. 3). Among individuals infected with HBV/E, 3 were co-infected with HBV genotype G and 2 with HBV genotype D (Fig. 4). Genetic diversity between HBV variants identified in 19 individuals infected with only one HBV genotype was in the range of 0.8%–3.6%, while individuals coinfected with two genotypes (HBV/G and HBV/D) showed higher variability ranging from 5.4%–7.1% (Table 2). The HBV sequence variants identified in the 2 individuals infected with genotype A3 strains were separated into 2 clusters in the phylogenetic tree (Fig. 1 and 2), indicating that both individuals were infected with 2 independent strains. Initial phylogenetic analysis showed strong intermixing of the HBV/E variants recovered from different residents (not shown). To visualize genetic interconnections between HBV variants and show variant frequencies in the population at the same time, we used a MJN [26]. Among 229 HBV variants, 210 (91.7%) were unique and 19 (8.3%) were found in more than one individual. The most frequent HBV sequence was shared by 10 residents of both villages; it was located in the center of the MJN and had the highest number of direct links to other sequences (Fig. 3). The majority of the high-frequency HBV variants were located in proximity to the center of the MJN. Among 19 HBV variants shared by more than one individual, 8 had a direct link to the most frequent variant in the center of the MJN (Fig. 3). These findings indicate a very close genetic relatedness between predominant and frequently shared HBV/E variants.

**Table 2:** Divergence within the HBV *S*-gene quasispecies.

Isolates	Age (years)	Gender	Community	HBV genotype	HBV Serotype	Number of Clones	Number of unique clones	% Divergence within quasispecies	Co-infecting HBV genotype
A32	30	M	1	E	*ayw4*	96	3	0.8	-
A51	66	M	1	E	*ayw4*	46	19	1.8	-
A58	33	M	1	E	*ayw4*	40	17	2.5	-
A67	60	M	1	E	*ayw4*	15	4	0.8	-
A84	30	M	1	E	*ayw4*	77	24	3.8	-
A85	15	M	1	E	*ayw4*	64	15	1.8	-
A100	19	M	1	E	*ayw4*	65	3	1.3	-
A112	21	F	1	E	*ayw4*	88	11	1	-
A178	5	F	1	E	*ayw4*	48	12	2.3	-
A227	18	M	1	E	*ayw4*	86	17	7.1 (2.8)	D
C16	24	M	2	E	*ayw4*	23	7	1.0	-
C19	25	M	2	E	*ayw4*	66	19	1.8	-
C8	25	M	2	E	*ayw4*	46	14	6.8 (1.8)	D
C98	12	F	2	E	*ayw4*	59	10	1.5	-
V5	23	M	1	E	*ayw4*	8	6	5.4 (2.0)	G
V16	21	F	1	E	*ayw4*	96	13	1.0	-
V30	22	M	1	E	*ayw4*	92	37	6.0 (0.8)	G
V64	27	M	1	E	*ayw4*	41	12	1.3	-
V67	19	F	1	E	*ayw4*	15	7	6.8 (1.5)	G
V100	26	M	1	E	*ayw4*	44	11	1.8	-
V137	20	M	1	E	*ayw4*	47	8	0.8	-
V157	24	M	1	E	*ayw4*	18	12	2.5	-
V103	22	M	1	A	*ayw1*	78	4	2	-
C3	24	M	2	A	*Ayw1*	45	12	2.5	-

*The percentage diversity in bracket represents the genetic distance observed when co- infecting viral genotypes was removed from the analysis. Gender: M = male, F = female. Location: Village 1 and village 2.

### Network of shared HBV sequences

**Figure 5:**
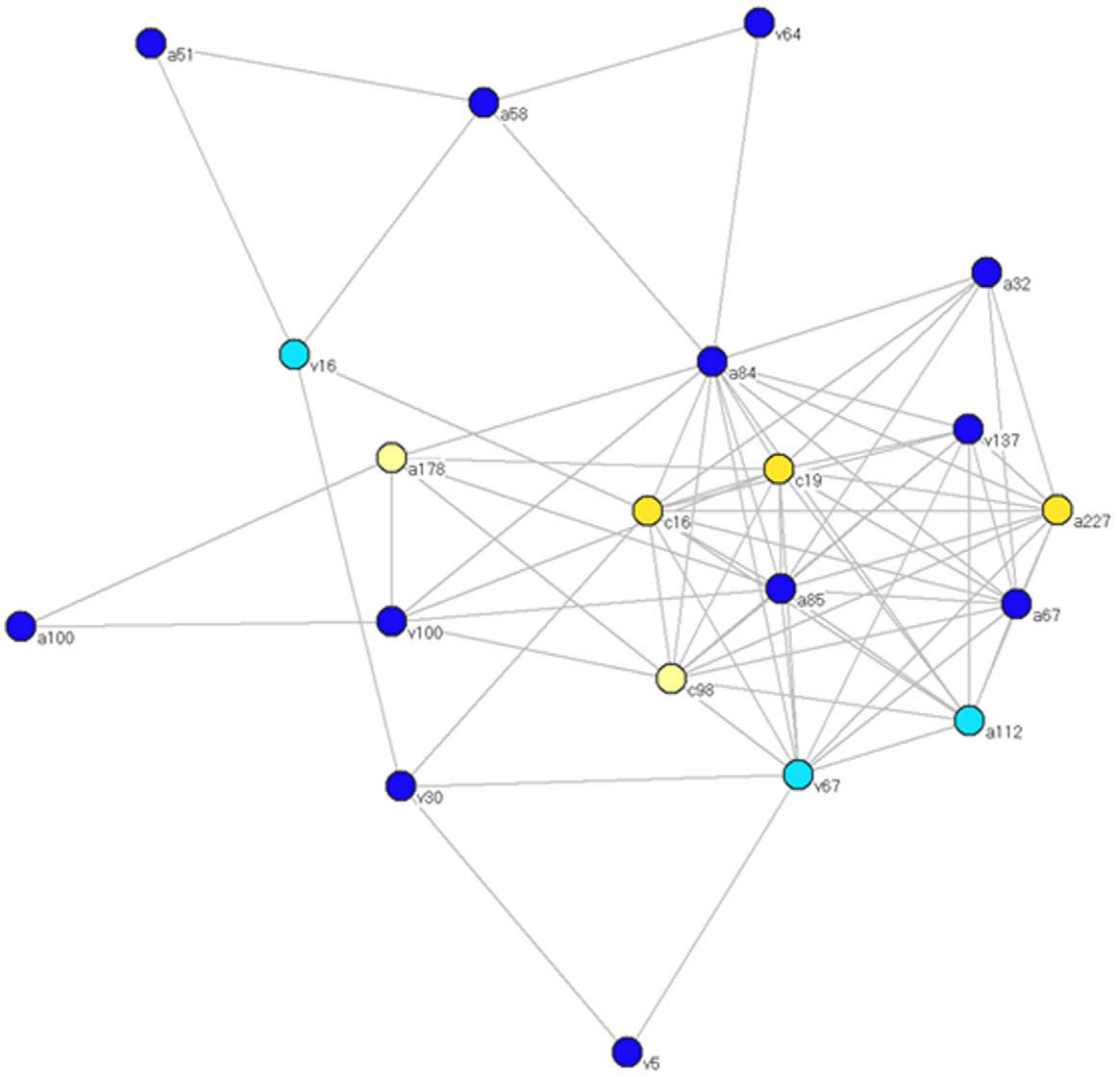
Network of individuals sharing identical sequences. Each node represents an individual and the link connects individuals that share at least one HBV variant. Blue nodes belong to village 1 (light for females, dark for males) and yellow nodes belong to village 2 (light for females, dark for males).

Interestingly, among the 22 HBV/E infected people only 2 were infected with unique strains, while the other 20 individuals were infected with at least one HBV variant shared by two or more residents. The quasispecies analysis identified 19 different sequences distributed among 20 HBV/E infected individuals, with each person sharing HBV sequences with 2 to 14 other people (Fig. 3). A network of shared HBV sequences was constructed (Fig. 5) using the distribution of HBV/E sequences among infected people. In this network, each node represents a person infected with the HBV/E strain and each link connects individuals sharing at least one HBV/E sequence. The network is very densely connected. Ten individuals, a67, a84, a85, a112, a227, c16, c19, c98, v67 and v137, representing a half of all nodes in the network, are completely interlinked, thus constituting the largest complete clique or the entirely interlinked component of the network.

The network and the largest clique included males and females of different ages residing in both villages (Fig. 5). Such extensive HBV strain sharing suggests very efficient HBV transmission between residents of both villages. This transmission seemed effective among both genders and people of a wide range of ages. Among 24 individuals tested for HBV quasispecies, 5 were females with the age ranged from 5 to 21 yr and 19 were males between 15 and 66 yr. All 5 females were infected with HBV/E strains shared with other persons. The linked females resided in both villages. Among 20 linked individuals, two were males, 60 and 66 yr old; both resided in the same village.

**Figure 6:**
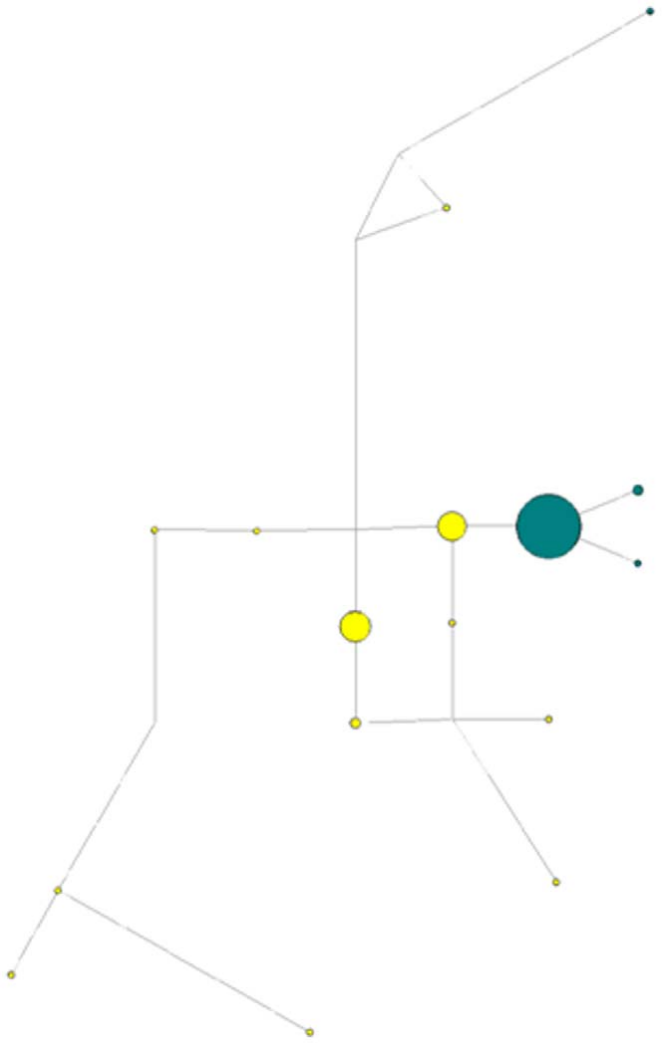
Median joining network of the intra-host S-gene sequence variants identified in 2 individuals infected with HBV/A3. Each node represents a single sequence variant. Each color represents a single individual. The size of the node reflects frequency of the corresponding variant in the population.

Among 24 individuals tested for HBV quasispecies, we identified two groups of siblings residing in the same village. Siblings a84 and a85 were found sharing HBV variants not only with each other but also with 13 and 9 other individuals, respectively (Fig. 5). Both were members of the largest clique in the HBV/E network. However siblings v5, v64 and v157 did not share any HBV/E variants with each other. Sibling v157 was not connected to the network. Siblings v5 and v64 were part of the network but did not have a direct link between each other and shared HBV/E variants only with 2 other individuals residing in the same village (Fig. 5). These observations suggest that HBV transmission between siblings was not the major route of transmission in these communities. Involvement of individuals with a wide range of demographic characteristics in the network of shared HBV/E sequences implies a complex pattern of transmission operating among residents of these 2 villages. This supposition is consistent with finding four HBV genotypes and numerous HBV variants circulating in the 2 communities. Interestingly, the quasispecies analysis of both HBV/A3 strains showed no coinfection with any other HBV strain found in this population (Fig. 6) despite a significant HBV prevalence and frequent coinfections between HBV/E strains.

### Selection pressures

Continuous circulation of HBV strains in closed communities may lead to the unique HBV adaptation under selection pressures acting specifically on their populations. Such selection pressures may be detected by determining d_N_/d_S_ ratios. Analysis of the d_N_/d_S_ values for each of the four ORFs of the 47 HBV/E isolates showed that the evolution of these HBV variants was driven predominantly by negative or purifying selection. Specifically, the mean d_N_/d_S_ for the *P*, precore/core, *X* and *S* genes were 0.390805 (95% CI, 0.3538–0.430356), 0.631286 (95% CI, 0.553566–0.715977), 0.880608 (95% CI, 0.745113–1.03199), and 0.638155 (95% CI, 0.537437–0.750901), respectively. However, five codons of the *P* gene, four codons of the precore/core gene, three codons of the *X* gene and one codon of the *S* gene showed statistically significant evidence of positive selection (Table 3).

**Table 3:** The number of negatively selected sites and specific sites under positive selection.

ORFs	Number of negatively selected sites	Positively selected sites	P-Value for positively selected sites
Polymerase	66	Codon 11	0.010608
		Codon 295	0.024116
		Codon 681	0.011953
		Codon 762	0.044131
		Codon 827	0.041043
PreC/Core	17	Codon 20	0.045301
		Codon 29	0.017184
		Codon 87	0.033245
		Codon 103	0.011595
X	8	Codon 76	0.036484
		Codon 127	0.006012
		Codon 129	0.028060
S	12	Codon 170	0.033047

A substitution within codon 762 of the *P* gene showed a statistically significant evidence of positive selection (p = 0.044). A non-synonymous substitution affected codon 768. These two codons encode amino acids at the anchor positions 2 and 8 within a potential HLA A23, HBV T-cell epitope [40], which is one of the most frequent HLA alleles in Nigeria and the West/Central Africa sub-region [16], [41], [42]. Although analysis of the HBV genotype A, C and D sequences retrieved from GenBank identified mutations within the same T-cell epitope, none of these mutations affected the anchor positions (data not shown). This finding suggests the HBV/E-specific adaptation to HLA-restricted immunological responses. The significance of the other sites under positive selection within the HBV/E genome is unclear.

### Geographic distribution of the most frequent HBV/E variant

The most frequent HBV/E sequence variant located in the center of the MJN (Fig. 3) was found to be identical to the HBV/E MRCA. A search of GenBank using BLAST yielded 160 nucleotide sequences identical to the HBV/E MRCA, 130 of which had associated information on their geographic location in Africa. These sequences were broadly distributed across West Africa (Benin, n = 2; Burkina Faso, n = 1; Gambia, n = 30; Ghana, n = 28; Guinea, n = 41; Mali, n = 3; Nigeria, n = 10; Togo, n = 2), Central Africa (Central African Republic, n = 1; Cameroon, n = 11) and Madagascar (n = 1).

### Time to the most recent common ancestor of HBV/E

The remote location of these 2 Nigerian villages suggests infrequent opportunities for HBV to be introduced to these communities. However, the identification of 4 HBV genotypes and numerous HBV/E variants implies more than one introduction of HBV. In order to evaluate the epidemic history of HBV/E in the villages we calculated the tMRCA for the HBV isolates identified in this study. A number of models were used which showed that the tMRCA for the HBV/E variants found in these communities existed ∼60 years ago (1952; 95% HPD: 1927–1970). However, it should be noted that analysis of sequences linked to a single date of collection could result in underestimating the true tMRCA. To place the tMRCA for these HBV/E isolates within the time frame for the entire HBV genotype E, the tMRCA was calculated for all HBV/E variants found in different countries of West/Central Africa in addition to those identified in this study. This calculation showed that the tMRCA for the entire HBV/E also existed ∼60 years ago (1948; HPD: 1924–1966). These results suggest that the HBV/E variants from the 2 communities originated at the same time as HBV/E strains circulating in the entire HBV/E crescent.

### Population dynamics of HBV/E

**Figure 7:**
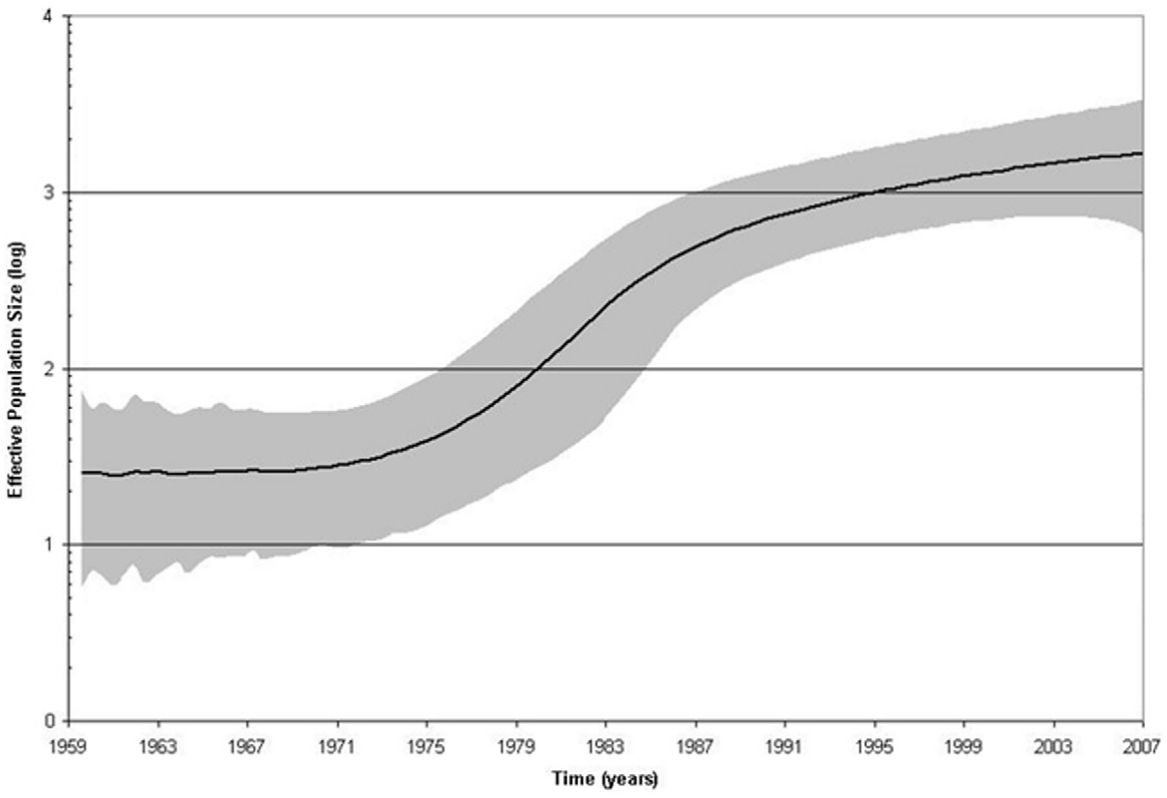
Bayesian skyline plot showing the epidemic history estimated from the Nigerian HBV genotype E dataset. The middle line is the median estimate of effective population size (log10) and the grey area shows the 95% highest posterior density intervals for this estimate. The most recent time is the time of collection for the most recently collected sequences (2007) and the oldest time shown is the lower limit of the 95% highest posterior probability density for the root height for all the sequences (1959).

The recent origin and broad distribution of the HBV/E variants in West/Central Africa suggests a rapid population expansion of HBV/E infections. Indeed, both Tajima's D and Fu's F_S_ tests rejected the null hypothesis of neutrality and constant population size (p = 0.0046 and 0.0009, respectively), suggesting the expansion of the HBV/E population. The star-like topology of the MJN constructed for the HBV/E quasispecies (Fig. 3) is consistent with this suggestion. Skyline plot analysis (Fig. 7) of HBV/E variants found in these 2 communities supported these observations and showed an increase in the effective number of HBV/E infections over time. Collectively, these findings indicate significant changes in the epidemiological processes affecting HBV/E infection over the last 50–100 years.

## Discussion

Analysis of serum specimens collected from two remote rural communities in North Central Nigeria revealed that ∼11% of their residents were actively infected with HBV. Although this high prevalence concurs with the rate of HBV infection of 10–40% found in other populations across Nigeria [13], [14], [15], [16], it is surprising that limited outside contacts did not shield these communities from HBV infection. Taking into consideration the isolation of these communities from the general population and the high prevalence of HBV infection, it is conceivable that only a few HBV strains could have been introduced and effectively transmitted among the residents. However, phylogenetic analysis of the *S*-gene sequences from 55 HBV isolates recovered in this study showed the presence of many HBV variants that belong to two genotypes, A and E (Fig. 1 and 2).

HBV/E was the most prevalent genotype, being detected among 96.4% of the isolates. Phylogenetic analysis showed that all the HBV/E variants were closely related to HBV/E strains circulating in West and Central Africa (Fig. 1 and 2). Detection of the most frequent HBV/E variant (Fig. 3) in many African countries strongly supports close genetic relatedness between HBV/E variants in these communities and other regions of Africa. These findings are in agreement with the previous observation of HBV/E in the majority of HBV-infected individuals in Nigeria [13]. Additionally, it confirms that Nigeria is part of the HBV/E crescent spanning countries from Senegal to Namibia [8], [11], [13], [43]. Only 2 (3.6%) isolates belonged to HBV/A3, and both preferentially clustered with the recently identified HBV/A3 isolates from Cameroon [44] rather than with HBV/A3 isolates from Gabon [45]. Cameroon could have been a possible source of HBV/A3 into Nigeria owing to the substantial volume of trade and intermarriage between these two neighboring countries (J.C.F. personal observation). None of the HBV/A in this study grouped with the African/Asian A1 or European/American A2 subgenotypes.

Of the 53 HBV/E isolates, 51 (96.2%) were predicted to belong to serotype *ayw4* and 2 (3.8%) to *ayw2*, while the 2 HBV/A3 isolates belonged to *ayw1*. These data are in disagreement with previous findings that all HBV/E from Africa belonged to serotype *ayw4* [13], [46], [47]. No evidence of recombination was found in the HBV/E *ayw2* isolates. The geographical distribution and frequency of HBV/E *ayw2* are not known.

Phylogenetic analysis of the whole-genome sequences (Fig. 2) did not reveal any village-specific clustering and showed extensive intermixing of these sequences with other HBV/E variants identified in different countries in Africa. Genetic diversity between the whole-genome sequences identified for HBV/E isolates circulating in the 2 communities was 2.1%, which is similar to the range found among all other HBV/E variants [8], [11], [43]. Such low genetic diversity among these local HBV/E isolates indicates that the virus might have only been recently introduced into these rural populations. Additionally, the presence of many HBV/E variants in the 2 communities and apparent intermixing with variants from other African countries suggest that all these HBV variants were introduced rather than having independently evolved in these communities. Because of limited contacts with outside populations, few opportunities were available for extraneous transmission, and, therefore, these HBV variants were most probably introduced to these villages simultaneously or within a very short timespan.

Analysis of HBV quasispecies from 24 residents of these 2 communities revealed that each individual was infected with many different HBV variants (Fig. 3). Identification of the genotype D and G sequences in 5 individuals (Fig. 4) adds another level of complexity to HBV populations circulating in the 2 communities. However, the consensus sequences identified by direct sequencing of PCR fragments (Fig. 1 and 2) did not accurately reflect the genotype complexity of the HBV population in each infected person. Among 22 residents infected with HBV/E, 20 shared HBV variants with 2–14 other individuals. Variant sharing was so extensive that a large network of shared HBV sequences linking 20 residents could be constructed (Fig. 5). Among these 20 residents, 10 were completely interlinked through shared HBV sequences to each other. Should variant sharing be considered as the proof of transmission [48], [49], this network reveals a very complex pattern of HBV transmission, which could be related to frequent infections with more than one HBV variant or to extensive superinfections with different HBV variants. The network contains male and female individuals of different ages residing in both villages, which suggests that HBV transmission occurred across both communities via a mode affecting the entire population.

The predominant mode of transmission leading to such significant HBV variant sharing is not known. Analysis of HBV quasispecies shared within 2 groups of siblings suggests that intra-familial transmission is not the only possible mode of transmission. These numerous HBV strains could have been maintained within the population through socio-cultural practices like facial or body scarification, traditional birth attendance and shaving by local barbers using unsterilized sharp instruments, all of which have the potential for the transmission of blood-borne pathogens and which have been associated with the transmission of human immunodeficiency virus (HIV) in Nigeria [50], [51].

Phylogenetic analysis also indicated a very close genetic relatedness between predominant and frequently shared HBV/E variants. These observations in conjunction with the star-like MJN topology suggest a very dynamic evolution of HBV/E variants found in these communities. The close connections between the HBV/E variants (Fig. 3 and 5) and frequent sharing of HBV sequences between individuals did not allow for a clear division of these HBV/E variants into separate strains, suggesting that the population of variants can be seen as a single swarm evolving among many hosts. Given the significant intermixing between the HBV/E variants found in this study and HBV/E variants identified in other African countries, together with the low heterogeneity of all these variants, this suggestion seems applicable to the entire HBV/E. The existence of this swarm of closely related HBV variants may reflect its recent origin followed by diversifying selection and adaptation to its particular transmission mode in the West/Central African HBV/E crescent.

The origin of HBV/E remains unclear. The very rare detection of HBV/E infections outside of Africa suggests that HBV/E became prevalent in the West/Central Africa only after the trans-Atlantic slave trade [8], [9], [10], [11], [12], [13], [43], [52]. This hypothesis of the recent HBV/E origin was supported by tMRCA analysis. The MRCA for the 47 HBV/E full-length isolates identified in this study was estimated to appear in ∼1952 (95% HPD: 1927–1970). Surprisingly, a similar tMRCA (∼1948; 95% HPD: 1924–1966) was estimated for the entire HBV/E. These findings indicate that the HBV/E variants identified in the 2 villages and in the entire HBV/E crescent in Africa have a similar time of origin.

Uncertainty in establishing the rate of substitutions for HBV may significantly affect estimates of tMRCA. Recently, tMRCA for HBV/E has been estimated to range between 30 to 1536 years depending on the used substitution rate [53]. The rate of 2.97×10^−4^ substitutions per site per year [34] used in the present analysis is most consistent with estimates made in several studies [54]. The analysis conducted here dated the HBV/E MRCA within a timeframe consistent with the hypothesis that modern HBV/E lineages emerged after the cessation of the trans-Atlantic slave trade [8], [11], [12], [13]. 

Although the modern HBV/E variants have a recent origin, the HBV/E variants were probably present long before the calculated tMRCA. Therefore, HBV/E could still have been introduced to the other parts of the world through the slave trade. The recent discovery of HBV/E variants among individuals of African descent in one isolated community in Colombia, South America [53] provides some support to this hypothesis. The coalescent analysis conducted in that study suggested that the closely related HBV/E variants found in that community originated from a single variant that existed a few years ago, thus indicating that only a single HBV/E strain was introduced to that community [53]. Considering its isolation and lack of contacts with persons traveling from Africa over the period of time exceeding the calculated tMRCA for these variants [53], it can be speculated that the ancestral HBV/E strain was introduced to that community many years ago, possibly even during the time of the slave trade.

The 2 isolated Nigerian communities in the current study were infected with and maintained a diverse population of HBV/E variants. This diversity is of the same degree as for all HBV/E isolates identified in different countries of Africa. Thus, HBV/E variants from Colombia [53] and the 2 communities in this study most probably had different evolutionary and epidemiological histories. Although the actual epidemiological and evolutionary processes leading to such a difference in the HBV/E sequence diversity are not known, it may be speculated that the conditions for massive introduction of multifarious HBV variants to the Nigerian communities did not exist when HBV was introduced to the Colombian community.

Strikingly, the estimated divergence dates for the identified HBV/E variants from the MRCA coincide with the period of intense mass public-health campaigns conducted in West/Central Africa. During 1967–1969, the World Health Organization mounted a large-scale program to eradicate smallpox and measles in West and Central Africa by arm-to-arm injections using jet injectors that have been recognized as presenting a significant risk for infection by blood-borne pathogens [55], [56], [57]. The geographical area stretching from Mauritania to the Congo (Zaire) river and from the Bight of Benin to the Sahara desert, covering 20 countries, constituted a single contiguous territory with ∼120 million inhabitants at that time. Between January 1967 and December 1969, one hundred million persons living in this belt were vaccinated against smallpox [57]. However, the mass vaccination practices were already adopted more than 50 years before the campaigns began. Mass inoculations against smallpox was carried out in the late 19^th^ century in Nigeria, Benin, Ghana, Guinea and Burkina-Faso that involved serial exchanges of blood and lymph [58], [59], [60], [61]. These could have constituted a route along which HBV/E was transmitted. It has been proposed that mass vaccination campaigns were associated with the dissemination of HBV/E in this region of Africa [55], [62]. In Egypt, unsafe injections used during nationwide campaigns against schistosomiasis between 1920 and 1980 have been associated with the country's high prevalence (>40%) of hepatitis C virus (HCV) infection [63], [64]. With HBV estimated to be ∼10 times more transmissible than HCV [62], [65], unsafe injections are a possible route of HBV/E transmission.

The data obtained in this study, although providing no direct indication on the actual events leading to the high HBV prevalence in the 2 communities, are consistent with massive transmission that resulted in rapid expansion of the HBV/E-infected population. In addition to the Bayesian coalescent, phylogenetic and MJN analyses described above, the Tajima's D and Fu's statistical tests [28], [29] provided support to this hypothesis. Analysis of the skyline plot (Fig. 7) for HBV/E variants identified in these 2 communities showed increase in the effective number of HBV/E infections ∼30–40 years ago. Identification of HBV/E variants identical in the S-gene sequence to the HBV/E MRCA in many countries in Africa also suggests a rapid HBV/E spread. The conditions facilitating such massive HBV/E expansion are not known. It is possible that HBV/E was the most prevalent genotype in this region of Africa before the expansion and/or the most adapted to the mode of transmission leading to such expansion. Analysis of selection pressures acting in the 2 Nigerian communities identified only few HBV/E sites under the positive selection (Table 3). Identification of a unique positively selected site at the anchor position of the potential T-cell epitope in the polymerase [40], [41], [42] supports a possible unique adaptation of HBV/E at the population level. All these findings suggest a dramatic shift in the epidemiological factors and evolutionary trends affecting the presentation of HBV/E in the West and Central Africa sub-region over the last century.
